# Dental Surgeons in the Army and Navy

**Published:** 1882-04

**Authors:** 


### DENTAL SURGEONS IN THE ARMY AND NAVY.

A movement is on foot aiming to secure the appointment of dentists in the army and navy. It is understood that a bill for this object will be presented to Congress at its next session, and that the subject is to be brought up at the meeting of the American Medical Association in 1882.

The need of dental services in the army and navy was first urged some thirty years ago. The matter has been twice laid before Congress since then, but without any action being taken. A dentist has been appointed to the Naval Academy at Annapolis with the rank of Assistant-Surgeon, and this is, we are told, all that has been done in the matter.

The want of dentists for our soldiers and sailors is very apparent. Sound teeth are one of the physical requirements for service, and it is only right that opportunities for preserving the teeth should be given.

On sea-voyages and in Western service it is quite impossible, as things now stand, that there should be any way of treating diseased teeth except by extraction. The testimony of several of the medical staff of the army and navy, as given in the *Times* recently, is to the effect that the need of dentists is much felt

There are a number of practical difficulties, however, in the way of creating and furnishing these services.

We have an army of only twenty thousand men, scattered in small garrisons throughout the country. It would hardly be feasible to appoint a dentist for each garrison, and the dental surgeon would have, therefore, to be a rather expensive itinerant. In the navy the difficulty would be still greater. In both branches there would, no doubt, be considerable opposition to admitting dentists to equal rank with medical men. For dentists have no right to call themselves medical or surgical specialists, unless they have gone through the same kind of education and training as that to which the gynecologist, laryngologist, or oculist subjects himself.

There is no doubt, however, that dentists are often needed and would be most useful in both army and navy. We should be glad to see the obstacles in the way of the introduction of their services there overcome. —*Medical Record.*

